# Spatial Guilds in the Serengeti Food Web Revealed by a Bayesian Group Model

**DOI:** 10.1371/journal.pcbi.1002321

**Published:** 2011-12-29

**Authors:** Edward B. Baskerville, Andy P. Dobson, Trevor Bedford, Stefano Allesina, T. Michael Anderson, Mercedes Pascual

**Affiliations:** 1 Department of Ecology and Evolutionary Biology, University of Michigan, Ann Arbor, Michigan, United States of America; 2 Department of Ecology and Evolutionary Biology, Princeton University, Princeton, New Jersey, United States of America; 3 Santa Fe Institute, Santa Fe, New Mexico, United States of America; 4 Howard Hughes Medical Institute, University of Michigan, Ann Arbor, Michigan, United States of America; 5 Department of Ecology and Evolution, Computation Institute, The University of Chicago, Chicago, Illinois, United States of America; 6 Department of Biology, Wake Forest University, Winston-Salem, North Carolina, United States of America; University of Texas at Austin, United States of America

## Abstract

Food webs, networks of feeding relationships in an ecosystem, provide fundamental insights into mechanisms that determine ecosystem stability and persistence. A standard approach in food-web analysis, and network analysis in general, has been to identify compartments, or modules, defined by many links within compartments and few links between them. This approach can identify large habitat boundaries in the network but may fail to identify other important structures. Empirical analyses of food webs have been further limited by low-resolution data for primary producers. In this paper, we present a Bayesian computational method for identifying group structure using a flexible definition that can describe both functional trophic roles and standard compartments. We apply this method to a newly compiled plant-mammal food web from the Serengeti ecosystem that includes high taxonomic resolution at the plant level, allowing a simultaneous examination of the signature of both habitat and trophic roles in network structure. We find that groups at the plant level reflect habitat structure, coupled at higher trophic levels by groups of herbivores, which are in turn coupled by carnivore groups. Thus the group structure of the Serengeti web represents a mixture of trophic guild structure and spatial pattern, in contrast to the standard compartments typically identified. The network topology supports recent ideas on spatial coupling and energy channels in ecosystems that have been proposed as important for persistence. Furthermore, our Bayesian approach provides a powerful, flexible framework for the study of network structure, and we believe it will prove instrumental in a variety of biological contexts.

## Author Summary

The relationships among organisms in an ecosystem can be described by a food web, a network representing who eats whom. Food web organization has important consequences for how populations change over time, how one species extinction can cause others, and how robustly ecosystems respond to disturbances. We present a computational method to analyze how species are organized into groups based on their interactions. We apply this method to the plant and mammal food web from the Serengeti savanna ecosystem in Tanzania, a pristine ecosystem increasingly threatened by human impacts. This web is unusually detailed, with plants identified down to individual species and corresponding habitats. Our analysis, which differs from the compartmental studies typically done in food webs, reveals that functionally distinct groups of carnivores, herbivores, and plants make up the Serengeti web, and that plant groups reflect distinct habitat types. Furthermore, since herbivore groups feed across multiple plant groups, and carnivore groups feed across multiple herbivore groups, energy represents a wider range of habitats as it flows up the web. This pattern may partly explain how the ecosystem remains in balance. Additionally, our method can be easily applied to other kinds of networks and modified to find other patterns.

## Introduction

Food webs, networks of feeding relationships in ecosystems, connect the biotic interactions among organisms with energy flows, thus linking together population dynamics, ecosystem function, and network topology. Ecologists have been using this powerful conceptual tool for more than a century [1]–[3]. One particularly relevant aspect of food webs is the subdivision of species into compartments or modules, a feature that has been proposed to contribute to food web stability by constraining the propagation of disturbances through a network [4]. In this definition, compartments are alternately referred to as modules, clusters, or “communities” [5], and are defined by high link density within groups and low link density between them. A large literature has considered the presence of compartments of food webs, with early work concluding that compartmentalization results primarily from habitat boundaries, not from dynamical effects [6], although continuing theoretical work has shown that compartmentalization can affect stability [7], [8]. One recent study shows that niche structure can result in compartmentalization [9], but the relationship between compartments and spatial habitat structure remains the strongest empirical pattern identified [10], [11].

Although compartmental structure may be significant at one scale of analysis, compartments alone do not account for much of the topological structure in food webs. Recent work with a probabilistic model considers a more flexible notion of groups, allowing link density to be high or low within any group or between any pair of groups [12]. Groups can thus represent compartments in the previous sense, but can also represent trophic guilds or roles [13], [14], sets of species that feed on, and are fed on, by similar sets of species. By fitting models of this type to data, the dominant topological pattern in the network can be found, which may include compartments, trophic guilds, or some combination of the two. The initial application of this model to empirical food webs from different ecosystems has revealed a predominance of trophic guilds rather than compartments [12].

Two major challenges limit the application of this model in resolving the group structure of food webs and interpreting its biological basis. First, most food webs have poor resolution of primary producers; plants in terrestrial systems and phytoplankton in aquatic ones are typically represented by a few nodes that are highly aggregated taxonomically. Some are aggregated at multiple trophic levels, e.g., the Coachella Valley web [15]; others aggregate only the primary producers, e.g., the El Verde rainforest [16], which identifies basal taxa as categories of plant parts. Another recently published Serengeti food web includes highly aggregated primary producers and varying levels of aggregation at other trophic levels [17]. Some webs that do include high resolution of plants include plant-herbivore bipartite networks, notably one lowland food-web from Papua New Guinea [18], and plant-insect-parasitoid “source webs” [19], [20]. Because primary producers form the base of the food web, high resolution in those groups can facilitate a much better understanding of how spatial organization and habitat type percolate up the web, and how higher trophic levels cut across the habitat structure at lower levels.

Second, some technical problems have hindered the use of probabilistic models in analyzing group structure. Early food web models served as null models for food web structure and were tested by generating model webs and comparing summary statistics against data from real webs [21], [22]. More recently, a more rigorous approach for measuring the goodness of fit of a model has been provided by maximum likelihood and model selection [12], [23]. Two problems still remain within this framework. One is technical: standard model-selection criteria are not applicable to “discrete parameters” such as group membership. The second problem is more fundamental: there are many almost equally good arrangements, and it is desirable to extract information not just from a single best arrangement, but also from the rest of the ensemble.

The Bayesian approach is gaining popularity in ecological modeling due to the philosophical and conceptual appeal of explicitly considering uncertainty in parameter estimation as well as its methodological flexibility [24]. This approach is especially well-suited for handling uncertainty in complex food web models, and allows us to overcome the limitations of the previous implementation of the group model. In network inference, there are only a few examples of complete Bayesian models [25], [26] and a few examples of MCMC for maximum-likelihood inference [27], [28], but Bayesian inference in phylogenetics has been long established [29], [30], and provides a clear methodological analogue.

In this paper, we address the group structure of a newly assembled food web for the large mammals and plants of the Serengeti grassland ecosystem of Tanzania by applying a computational approach to the identification of groups based on Bayesian inference. We specifically ask whether the structure that emerges reflects the underlying spatial dimension, as delineated by the different plant communities that characterize different sub-habitats within the ecosystem, or whether it is determined by trophic dimensions in the form of species guilds that share functional roles.

The Serengeti has been studied as an integrated ecosystem for almost five decades [31]–[33], and because of widespread popular familiarity with the consumer-resource dynamics of lions, hyenas, wildebeest, zebra and grasses, it provides a strong intuitive test for probabilistic food web models. Furthermore, all the primary producers in this Serengeti web are identified to the genus or species level. The plant diversity encompasses a number of distinct grass, herb, and woody plant communities on different soils and across a rainfall gradient [34]. This well-documented structure allows us to examine the extent to which habitat structure defines network topology at multiple trophic levels. Although not yet a comprehensive community web, with the addition of more taxa, such as those in another recently published Serengeti web [17], this data set can become the most highly-resolved terrestrial web available.

## Results

### The Serengeti Food Web Data Set

We compiled the Serengeti food web from published accounts of feeding links in the literature [34]–[47] along with some links known from personal observation. With a few exceptions, the taxa included are large mammalian carnivores and herbivores and the plant diets of the herbivores. In its current form it is not a comprehensive community web, nor does such a terrestrial web yet exist. Another recently published Serengeti food web is largely complementary, containing many bird, mammal, and invertebrate species not included here, but without high resolution of plants [17]. We have not included invertebrates (insects and parasitic helminths) or birds, but are adding data for these groups for future studies.

The compiled food web (Tables S1 and S2) consists of 592 feeding links among 161 species (129 plants, 23 herbivores, and 9 carnivores). 507 of the links are herbivorous, and 85 are predatory. The fraction of all possible links (connectance, 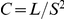), ignoring all biological constraints, is equal to 0.023. We attribute the low connectance, as compared to other existing food-web data sets, to the high taxonomic resolution of the plant community.

### Performance of Model Variants

We compared marginal likelihood estimates of different model variants to determine which one best describes the Serengeti food web (see Methods). First, we find unequivocal support for the use of group-based models in describing the Serengeti food web, as compared with simple null models that ignore group structure, either by treating each species as its own group or by combining all species into a single group (Table 1). We also find that a flexible group model that allows for high or low connectance between and within groups vastly outperforms a compartmental model that restricts between-group connectance to be lower than within-group connectance, with a posterior odds ratio (Bayes factor) of 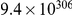 against the compartmental model.

**Table 1:** Marginal likelihood estimates and Bayes factors relative to best model.

Group model	Partition prior	Link prior	Log MLE	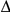 MLE	Bayes factor
One group	—	Uniform	−2828.60	−1556.82	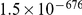
161 groups	—	Beta	−2828.60	−1556.82	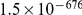
161 groups	—	Uniform	−17967.07	−16695.28	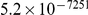
Compartmental groups	Dirichlet process	Beta	−1978.76	−706.97	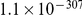
Flexible groups	Uniform	Uniform	−1710.83	−439.04	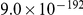
Flexible groups	Uniform	Beta	−1404.32	−132.53	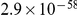
Flexible groups	Dirichlet process	Uniform	−1455.32	−183.54	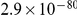
Flexible groups	Dirichlet process	Beta	−1271.78	0	1

Additionally, the use of flexible priors vastly improves the fit of the basic model, for both link probability parameters and network partitions. The model variant with beta prior for link probabilities and Dirichlet process prior for partitions performed best. Next, in order, were (1) the model with beta link probability prior and uniform partition prior, (2) the model with uniform link probability prior and Dirichlet process partition prior, and (3) the model with both uniform priors. The strongest variant surpassed its closest competitior by 133 units of (natural) log-likelihood, corresponding to a posterior odds ratio of 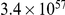 against the worse one, and surpassed the model with both uniform priors by 439 units of log-likelihood, a posterior odds ratio of 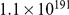. In all cases, 95% confidence intervals on the marginal likelihood estimates were less than one unit of log-likelihood, far less than the differences between models. Given this unequivocal support, we consider results only from the best model variant.

### Identification of Model Parameters

**Figure 1:**
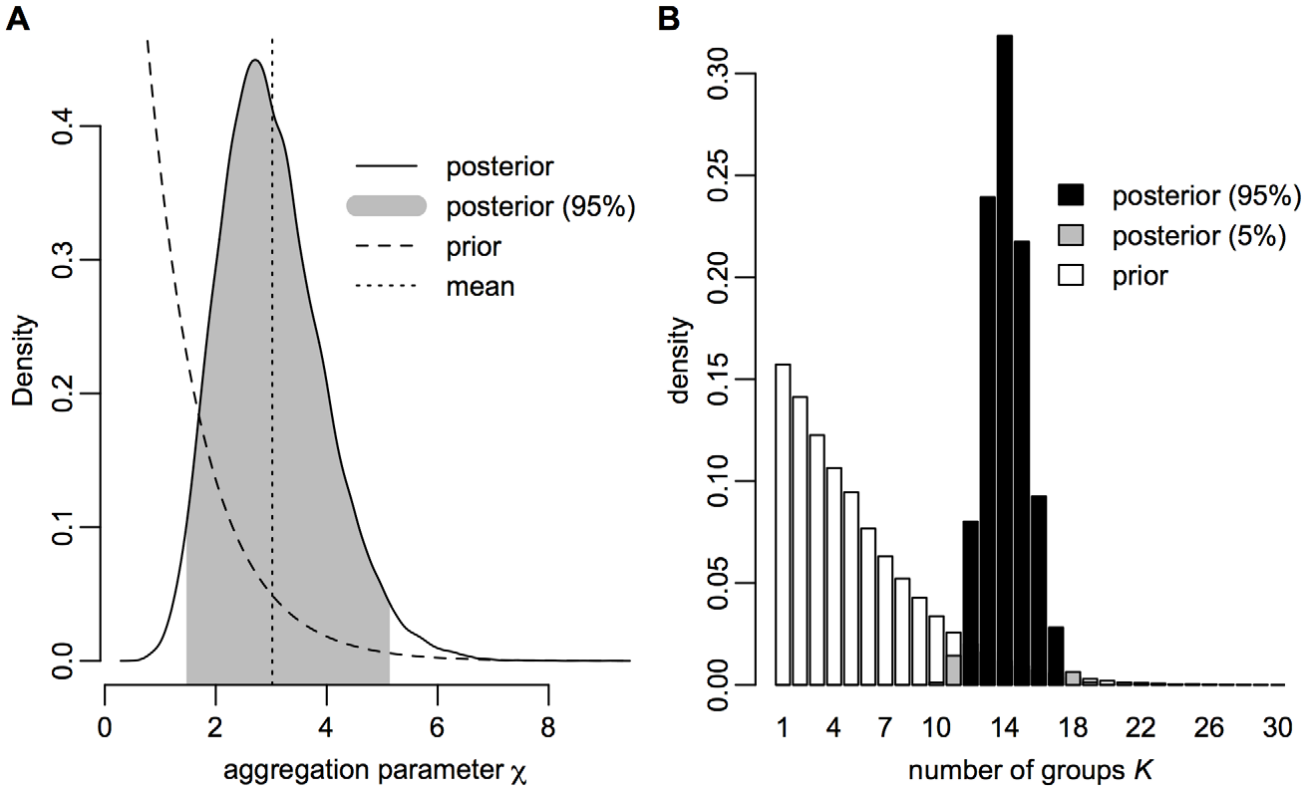
Posterior distributions and prior expectations of aggregation parameter 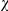 and group count 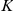.

We used samples from the posterior distribution to summarize model hyperparameters controlling link probabilities and partitions. The posterior mean number of groups 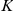 is 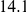 (95% credible interval 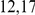), and the mean value of the Dirichlet process parameter 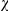 is 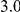 (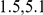) (Figure 1). The prior expectation of 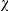 was 1.0 and the prior expectation of 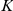 was 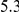. The finding of posterior values substantially greater than prior values strongly supports the presence of detailed group structure in the Serengeti food web.

Mean values for beta distribution parameters are 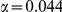 (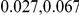) and 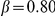 (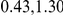) (Figure S2). The corresponding beta prior has support concentrated near 0, since most species do not feed on most other species (Figure S3).

### Consensus Partition

**Figure 2:**
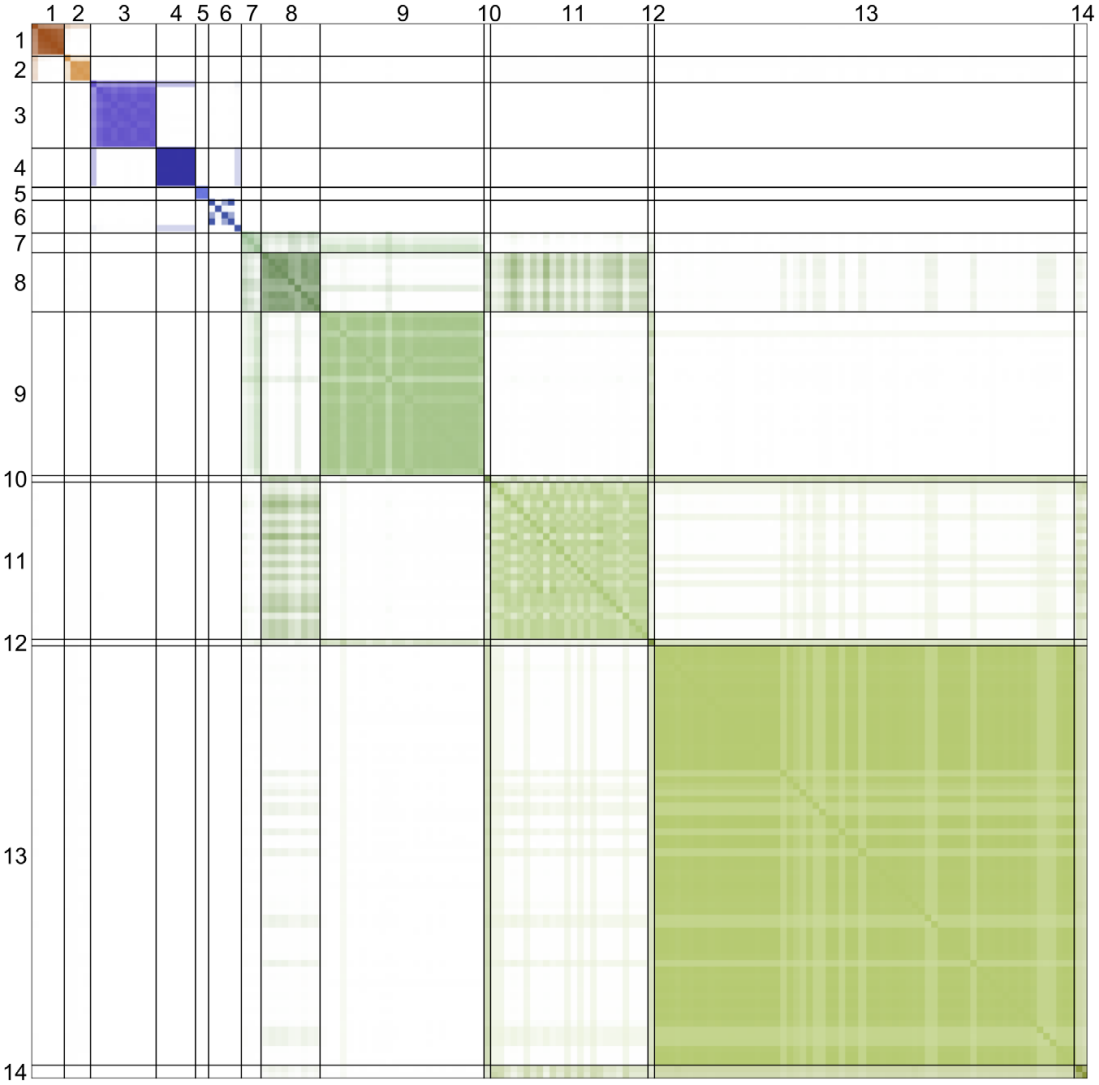
Affinity matrix. Species are identically ordered top to bottom and left to right according to the consensus partition as listed in Table 2. Hue indicates group identity; color saturation indicates the fraction of partitions in which species occupy the same group. Note that this image conveys information about group membership, not network connectivity.

The posterior output includes 30,000 partitions of the Serengeti food web into groups, nearly all distinct from each other. One partition appears 6 times; two partitions appear 3 times; 14 partitions appear 2 times, and the rest appear only once. For the sake of interpretation, we formed a consensus partition (Table S3) of 14 groups from the affinity matrix (Figure 2), which represents the fraction of partitions in all posterior samples in which pairs of species appear in the same group. On average, the consensus partition differs from sampled partitions by 5.6%, calculated as the fraction of species pairs that are assigned to the same group in one partition but to different groups in the other. By comparison, on average, individual sampled partitions differ from other sampled partitions by 7.9%. In addition, every sampled partition differs on average from the others by more than the consensus partition does, indicating the value of the consensus approach.

### Groups Identified in the Serengeti Food Web

**Figure 3:**
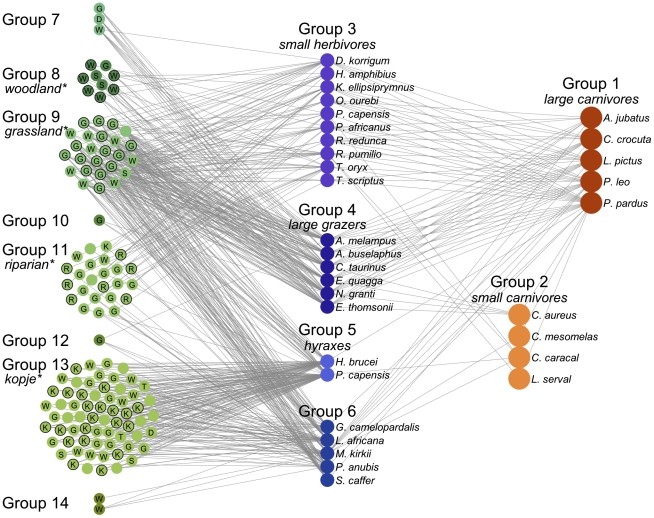
The Serengeti food web. The network is shown organized and colored by group according to the consensus partition listed in Table 2, and arranged by trophic level from left (plants) to right (carnivores). Plants are identified by the first letter of identified habitat type, if available: (G)rassland, (W)oodland, (R)iparian, (K)opje, (S)hrubland, (T)hicket, and (D)isturbed. Plant groups are labeled by significantly overrepresented habitat types, and species assigned to the overrepresented type are labeled with black borders. An interactive version of this figure will be made available at http://edbaskerville.com/research/serengeti-food-web/.

**Figure 4:**
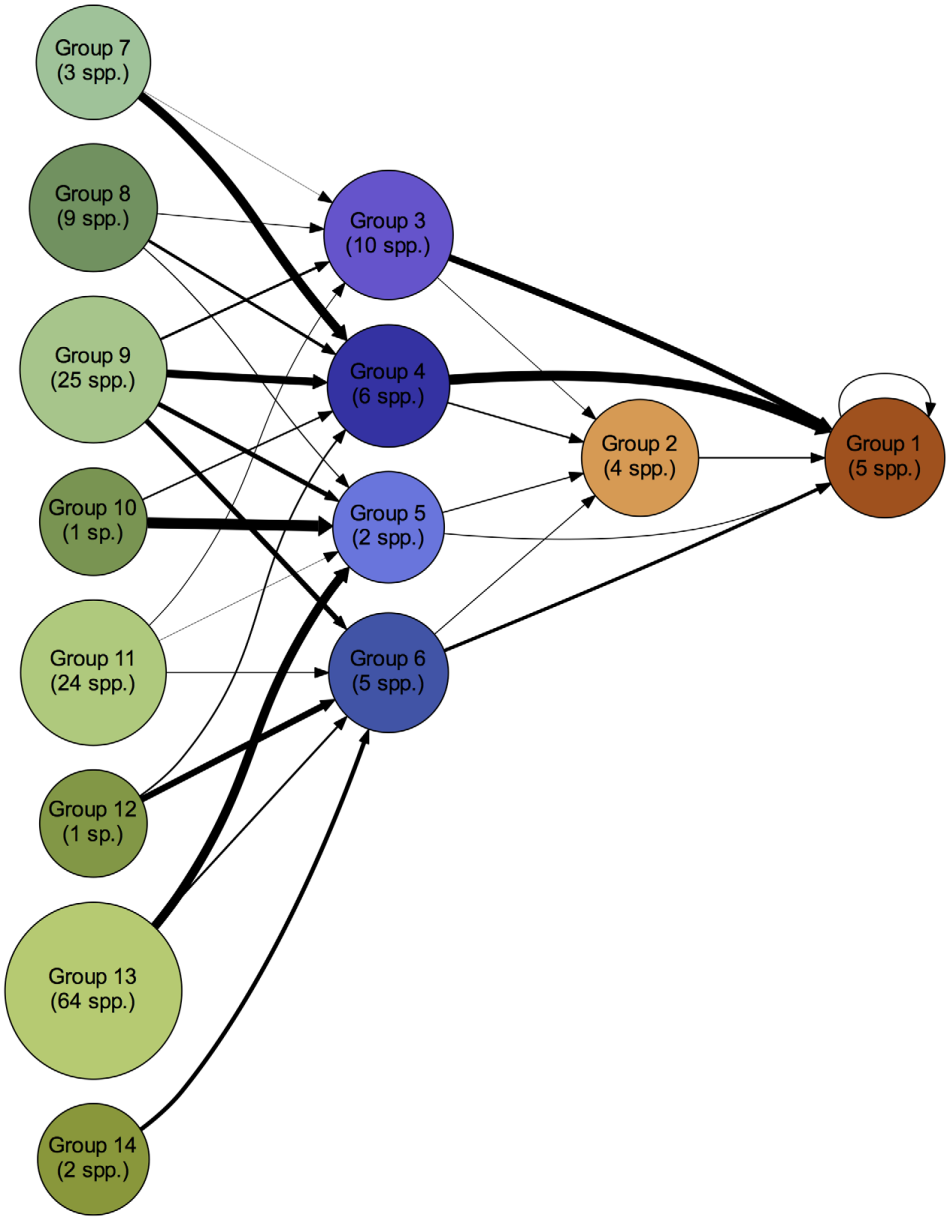
Network layout of aggregated groups. Nodes in the network are aggregated and colored by group according to the consensus partition listed in Table 2, and arranged by trophic level from left (plants) to right (carnivores). Line thickness indicates the link density between groups. Node area increases with the number of species in a group.

The groups identified in the Serengeti food web in the consensus partition contain trophically similar species, with all groups restricted to a single trophic level (plants, herbivores, or carnivores). The consensus partition, with 14 groups, is shown in Table 2. The partition includes 2 groups of carnivores (groups 1–2), 4 groups of herbivores (groups 3–6), and 8 groups of plants (groups 7–14). On average, plant groups contain more species than herbivore and carnivore groups (16.1, 5.8, and 4.5, respectively). As evident in the affinity matrix, the carnivore and herbivore groups are well-defined, including several individual species or pairs of species with distinct diets. Plant groups demonstrate mild overlap, indicating a partially hierarchical relationship between smaller groups and larger groups. Figures 3, 4, and S1 show three alternate views of the food web, organized by the 14 -group consensus partition. Except for carnivore group 1, there are no connections within groups, and partitions are defined by targeted, directed connections between specific pairs of groups. For actual link densities between groups in the consensus partition, see Table S4.

**Table 2:** Groups identified in the Serengeti food web using a 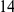-group consensus partition.

Group 1	Acinonyx jubatus, Crocuta crocuta, Lycaon pictus, Panthera leo, Panthera pardus
Group 2	Canis aureus, Canis mesomelas, Caracal caracal, Leptailurus serval
Group 3	Damaliscus korrigum, Hippopotamus amphibius, Kobus ellipsiprymnus, Ourebia ourebi, Pedetes capensis, Phacochoerus africanus, Redunca redunca, Rhabdomys pumilio, Taurotragus oryx, Tragelaphus scriptus
Group 4	Aepyceros melampus, Alcelaphus buselaphus, Connochaetes taurinus, Equus quagga, Nanger granti, Eudorcas thomsonii
Group 5	Heterohyrax brucei, Procavia capensis
Group 6	Giraffa camelopardalis, Loxodonta africana, Madoqua kirkii, Papio anubis, Syncerus caffer
Group 7	Digitaria scalarum, Dinebra retroflexa, Hyparrhenia rufa
Group 8	Chloris gayana, Combretum molle, Digitaria diagonalis, Duosperma kilimandscharica, Eragrostis cilianensis, Microchloa kunthii, Sporobolus festivus, Sporobolus fimbriatus, Sporobolus spicatus
Group 9	Acacia tortilis, Andropogon greenwayi, Aristida spp., Balanites aegyptiaca, Bothriochloa insculpta, Brachiaria semiundulata, Croton macrostachyus, Cynodon dactylon, Digitaria macroblephara, Eragrostis tenuifolia, Eustachys paspaloides, Grewia bicolor, Harpachne schimperi, Heteropogon contortus, Hibiscus spp., Hyparrhenia filipendula, Indigofera hochstetteri, Panicum coloratum, Panicum maximum, Pennisetum mezianum, Sida spp., Solanum incanum, Sporobolus ioclados, Sporobolus pyramidalis, Themeda triandra
Group 10	Pennisetum stramineum
Group 11	Acacia seyal, Acacia xanthophloea, Andropogon schirensis, Chloris pycnothrix, Chloris roxburghiana, Crotalaria spinosa, Cymbopogon excavatus, Digitaria milanjiana, Digitaria ternata, Echinochloa haploclada, Eragrostis exasperata, Euphorbia candelabrum, Hyperthelia dissoluta, Kigelia africana, Lonchocarpus eriocalyx, Olea spp., Panicum deustum, Panicum repens, Phragmites mauritianus, Psilolemma jaegeri, Sarga versicolor, Setaria pallidefusca, Setaria sphacelata, Typha capensis
Group 12	Acacia senegal
Group 13	Abutilon spp., Acalypha fruticosa, Acacia robusta, Achyranthes aspera, Albizia harveyi, Albuca spp., Allophylus rubifolius, Aloe macrosiphon, Aloe secundiflora, Blepharis acanthodioides, Capparis tomentosa, Pennisetum ciliare, Cissus quadrangularis, Cissus rotundifolia, Commelina africana, Commiphora merkeri, Commiphora schimperi, Cordia ovalis, Croton dichogamus, Cyperus spp., Cyphostemma spp., Digitaria velutina, Diheteropogon amplectens, Emilia coccinea, Eragrostis aspera, Eriochloa nubica, Ficus glumosa, Ficus ingens, Ficus thonningii, Grewia fallax, Grewia trichocarpa, Heliotropium steudneri, Hibiscus lunariifolius, Hoslundia opposita, Hypoestes forskaolii, Iboza spp., Indigofera basiflora, Ipomoea obscura, Jasminum spp., Kalanchoe spp., Kedrostis foetidissima, Kyllinga nervosa, Lippia ukambensis, Maerua cafra, Ocimum spp., Pappea capensis, Pavetta assimilis, Pavonia patens, Pellaea calomelanos, Phyllanthus sepialis, Pupalia lappacea, Rhoicissus revoilii, Sclerocarya birrea, Senna didymobotrya, Sansevieria ehrenbergii, Sansevieria suffruticosa, Solanum dennekense, Solanum nigrum, Sporobolus pellucidus, Sporobolus stapfianus, Tricholaena teneriffae, Turraea fischeri, Ximenia caffra, Ziziphus spp.
Group 14	Boscia augustifolia, Commiphora trothae

### Habitat Signature and Food-Web Structure

Overall, plants of the same habitat type are significantly more clustered in groups than random according to weighted Shannon entropy. (Lower values of weighted entropy indicate higher levels of clustering; see Methods.) Mean weighted entropy across all posterior partitions is 1.25 , compared to a randomized mean value of 1.39 (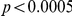).

Furthermore, the four largest plant groups reflect significant overrepresentation of four different habitat types, and either significant underrepresentation or no significant signal for other habitat types. In group 13, kopje plants are significantly overrepresented, comprising 36.7% of the group, compared to a random expectation of 18.1% (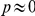). Group 9 contains 60.4% grassland plants compared to a random expectation of 41.5% (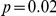), and includes 40.4% of individual species records in the plot data. All of the identified riparian species occur in group 11, comprising 31.8% of the group, compared with a 6.3% random expectation (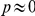). Finally, woodland plants comprise 66.7% of group 8, compared with a random expectation of 25.6% (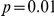). This result holds across all individual sampled partitions in the posterior output; each one includes four different groups with significant overrepresentation of kopje, grassland, riparian, and woodland habitat.

Plant groups are coupled by groups of herbivores, which are in turn coupled by groups of carnivores. Large migratory grazers (group 4, wildebeest, zebra, and gazelles) feed plant groups that include the dominant grass species in the ecosystem (group 9), predominantly riparian species (group 11), and a mixture of woodland species (*Combretum molle*, *Digitaria diagonalis*, *Duosperma kilimandscharica*, and others) and less common species (group 8). Group 7 represents a specific case where very high trophic similarity brings two spatially separate plants into the same group. *Hyparrhenia rufa* is found mainly in the north, and is a significant component of zebra and wildebeest diets during the dry season; in contrast, *Digitaria scalarum* dominates much of the plains and is eaten in large amounts by migrants during the rainy season when their nutritional needs are at a maximum due to calving and lactation. However, they are grouped together because of their mutual inclusion in the diets of the migratory species. Herbivores feeding in the longer grasslands, woodlands and in riparian habitats (group 3) couple groups 9 and 11. The hyraxes (group 5) and group 6 (giraffe, elephant, buffalo, and others) couple group 13, which bears a strong kopje signature, to groups biased toward other habitats. At the highest trophic level, the large carnivores (group 1) integrate across all the herbivore groups; smaller carnivores (group 2) show more specialized diets, reflecting the more distinct habitats in which they are usually found.

## Discussion

### Spatial Guilds in the Serengeti Food Web

In order to analyze the group structure of the Serengeti food web, we used a flexible Bayesian model of network structure that includes no biological information aside from a set of nodes representing species and links representing their interactions. The groups that emerge from an otherwise blind classification of species make remarkable biological sense, and moreover reveal detailed patterns between habitat structure and network topology that expert intuition alone cannot. Species are divided into trophic guilds that reveal a strong relationship between the habitat structure of plant, herbivore, and carnivore groups and the structure of the network. At the coarsest scale, the groups in the Serengeti food web correspond to carnivores, herbivores, and plants. The further subdivisions that emerge within the trophic levels reveal connections between habitat types and feeding structure. This deeper analysis is made possible by high resolution at the plant level along with information about the habitat occupancy of different plants. Since different habitat types occupy distinct spatial locations in the Serengeti, the group structure thus reflects in part the flow of energy up the food web from different spatial locations, with herbivores integrating spatially separated groups of plants, and carnivores integrating spatially widespread herbivores. A priori, it was not clear precisely what kind of group structure would emerge in the Serengeti web from the use of the group model. In general, the more complex the web, the more useful these methods will be in helping to disentangle the complexity.

The food web presented here included only plants and mammals, but we hypothesize that the general conclusions will be largely robust to the addition of more species. Although the addition of birds, reptiles, invertebrates, and pathogens will likely add a significant number of new groups, they should not significantly modify the derived structure for mammals, since the insect-bird links reflect an almost parallel food web. To the extent that insect herbivores further differentiate plants, plant groupings may be affected, but we expect that the larger tendency for groups to reflect habitat structure will remain.

Recently, interesting theoretical and empirical work has highlighted the relationship between observed patterns of food-web structure and energy flow that seemingly relates to the trophic guild structure in the Serengeti. Rooney and colleagues [48] give evidence that real ecosystems may be dominated by nested sets of fast and slow “energy channels, ” each of which represents a food chain of trophic guilds. They suggest that this pattern may have a strong stabilizing effect, based on theoretical work by McCann on spatially coupled food webs [49]. The group structure for the Serengeti web that emerges from our analysis supports a pattern of spatial coupling at multiple trophic levels: the grasslands have very high turnover rates compared to those of the kopjes and woodlands. This suggests a similar pattern of fast and slow energy channels to those described by Rooney and colleagues, with fast energy flow up through the highly seasonal but very productive grasses of the short-grass plains. These are almost completely consumed by wildebeest and zebra during their peak calving season, which are then in turn consumed by large predators (lions and hyenas). Although the migratory species of wildebeest and zebra form a crucial and major component of the diet of the predatory species, their high abundance and presence in open habitat places them at a lower per capita risk of predation. In contrast, the resident herbivore species living on kopjes and in the woodlands reproduce at slower rates and are consumed at higher per capita rates by large carnivores during the time when the carnivores are unable to feed on migratory wildebeest and zebra.

These patterns emerge directly from the topology of the food web without being explicitly labeled as different habitats upfront as was done in previous empirical work [48], showing that topological analysis can reveal structures that may be very significant for food-web dynamics. They are subtly different, however, from the proposed pure fast and slow chains, in that they incorporate the migration of the keystone species in the ecosystem, so the fastest energy chain is seasonally ephemeral and may only operate for three to four months in any year. We suspect that even within the sub-habitats of kopjes and woodlands there are similarly nested faster and slower chains that involve species for which we are still collating data (e.g., birds, small mammals, and insects). More generally, we see the identification of important structures in empirical food webs via probabilistic models as important for grounding future investigations into the relationship between structure and dynamics in empirical pattern.

### Bayesian Analysis of Food-Web Structure

In this paper, we used a probabilistic model to analyze the structure of a single food web, an approach we have seen in only one other study based on a probabilistic version of the niche model [28] (see supporting text S1 for more discussion of probabilistic modeling of food webs). This approach has proved fruitful in Bayesian phylogenetics, where the combinatorial challenges are similar. Moreover, we view the group model as only a starting point for richer modeling efforts to help identify relevant processes that influence the structure of ecological communities.

In fact, the Bayesian approach described here provides a powerful general framework for encoding hypotheses about the structure of food webs and comparing models against each other, and we see it as a natural next step in the current trend of representing food-web models in a common way. Simple abstract models such as the niche model and the group model used here act as proxies for the high-dimensional trait space that determines feeding relationships in an ecosystem. The identification of actual traits that correspond to groups (or niche dimensions) is another valuable direction, so far followed primarily by finding correlations between compartments/groups [11] or niche values [22] and traits such as body size or phylogenetic relatedness. A more sophisticated, rigorous approach is to directly incorporate such traits into the probabilistic models themselves, either as predictors or as informed Bayesian model priors. Although the current work does not employ that approach, the results from the habitat analysis suggest that including additional information directly in the model would be a valuable approach.

The use of flexible, hierarchical priors for model parameters is another useful innovation of the Bayesian framework. The number of groups identified by the model increases dramatically with the use of a flexible beta prior distribution for link probability parameters. In that model variant, we effectively introduce two degrees of freedom to the model (the beta distribution parameters) but dramatically reduce the effective degrees of freedom of the link probability parameters. Note that we penalize parameters by using the marginal likelihood for model selection, so that the model selection represents a balance between goodness of fit and model complexity. Moreover, this structure makes intuitive sense: since most link probability parameters are simply zero, they should not be penalized. An alternate approach is to remove and add parameters to the model, but this hierarchical technique is much easier to implement in practice.

Advanced Markov-chain Monte Carlo methods make it possible to accurately estimate marginal likelihoods for probabilistic network models. Unlike information criteria such as AIC or BIC, an accurate estimate of the marginal likelihood provides a direct measurement of goodness of fit that takes into account the degrees of freedom in a model without making any asymptotic assumptions about parameter distributions [50], and can handle discrete parameters such as partitioning into groups that are not properly handled by AIC and BIC.

Additionally, the Bayesian approach also serves as a means to avoid fundamental issues inherent in network models with a large parameter space. One recent study has shown that, even in relatively small networks, a large number of good solutions exist for the standard modularity criterion [51], [52]. A maximization algorithm is thus guaranteed to find a single local maximum of many—possibly even the best one, but certainly not one that captures the full range of good solutions. This problem arises whether the quantity to be maximized is a heuristic such as modularity or a likelihood value. The group model and other parameter-rich models presumably suffer from similar degeneracy problems. In the present case, we find that nearly every partition sampled from the posterior distribution is unique. Although MCMC sampling cannot reproduce the full posterior distribution, it is an important step in the right direction. Philosophical arguments aside, one of the main reasons for maximizing likelihood or modularity is simply that a single solution is far more tractable than a distribution. The consensus partitioning heuristic used here is an attempt to find a single partition that represents the posterior distribution reasonably well for the sake of interpretation and presentation (see Methods). More sophisticated approaches to collapsing partitions will be welcomed. However, since the Bayesian approach provides direct access to uncertainty in the form of the posterior distribution, quantitative analyses should be done across the whole distribution, and we follow that approach here.

### Conclusion

The group model, based on the simple notion that groups of species may have similar feeding relationships to other groups, reveals that trophic guilds are the topologically dominant type of group in the Serengeti food web. The model also reveals an interesting relationship between habitat structure and network structure that corroborates recent ideas on spatial coupling in food webs. A theoretical study with a dynamical model suggests that this type of structure may contribute to ‘stability’ in the sense of the persistence of species [49]. Now, by using group structures directly inferred from empirical webs, we can better guide investigations into the relationship between structure and various aspects of stability, for example robustness to secondary extinctions [53], [54]. Although the Bayesian modeling approach is not new to network analysis in general [25], [26], it remains relatively rare. The Bayesian group model, and, more importantly, the general framework for modeling and model selection, naturally extend to other kinds of biological networks, such as metabolic and regulatory networks [55] and networks describing other ecological interactions such as pollination [56]. We advocate this framework as a way to build stronger ties between hypothesis formulation, model building, and data analysis.

## Methods

### The Bayesian Group Model

In this work, we use Bayesian probabilistic models to analyze food webs; for a general introduction to the Bayesian modeling approach and details on the specific models used here, please see supporting text S1. We employ a generative model based on groups that treats the food-web network, represented as the presence or absence of each possible feeding link, as data. The group model [12], known as a stochastic block model in the statistical literature [57], was previously treated in a maximum-likelihood framework. In a Bayesian framework, both data and model parameters are treated probabilistically, making the object of inference a posterior distribution over model parameters rather than a point estimate. For a general overview of Bayesian inference, see section 3 of supporting text S1.

The group model (supporting text S1, section 2) divides species into some number of groups 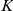, thus determining a *partition*. All possible links between any pair of groups are assigned the same probability of existing, 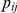, for consumer group 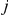 and resource group 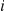. If a between-group link probability 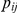 is close to one, then there are likely to be many links with a species from group 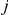 feeding on a species from group 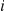. A highly compartmental network can be generated by having lower between-group link probabilities 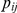 (for 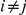) than within-group link probabilities 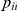.

### Priors and Model Variants

In general, priors may incorporate informed knowledge about the system, but in this case we simply use them to encode different variants of the same basic model. We use two distributions for partitions and two distributions for link probabilities, which are combined to form four different model variants. We also consider several null models for comparison.

#### Partition prior

For partitions, we employ two prior distributions: (1) a uniform distribution and (2) a distribution generated by the Dirichlet process, sometimes referred to as the “Chinese restaurant process” [58]. Alternative (2) is controlled by an aggregation parameter 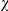 that is in turn drawn from an exponential distribution with mean 1. The uniform distribution assigns equal prior probability to each possible partition, irrespective of the number of groups. Because there are far more ways to partition the network at an intermediate, but relatively high, number of groups, the uniform prior implicitly biases the model toward that number. For example, for a network of 100 nodes, there is an *a priori* expectation of 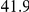 groups. In contrast, the hierarchically structured Dirichlet process prior provides flexibility via the aggregation parameter 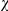. When 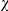 is large, partitions tend to have many small groups; when 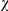 is small, partitions tend to have fewer groups, with a skewed group-size distribution. See section 3.1 of supporting text S1 for mathematical details and a fuller discussion.

#### Link probability prior

The two alternative prior distributions used for link probabilities 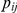 are (1) a uniform distribution between 0 and 1, and (2) a beta distribution with shape parameters 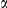 and 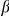, which are in turn governed by exponential distributions with mean 1. With 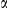 and 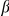 fixed at their means, alternative (2) reduces to a uniform distribution; at other values, the distribution may take a uniform, convex, concave, or skewed shape. The second alternative is thus structured hierarchically, with exponential *hyperpriors* for 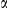 and 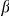 governing the beta prior for link probabilities 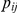. For more details, see section 2.1 of supporting text S1.

#### Null models

We also consider two simple models without groups as null comparisons: (1) a directed random graph model (i.e., one group) with a uniform prior on a single link probability parameter 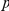, and (2) a fully parameterized model, with each species in its own group, and a 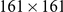 link probability parameter matrix 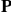, also with a uniform link probability prior.

Finally, in order to explicitly restrict the model to detecting compartmental structure, we also consider a modification that requires all between-group link probabilities 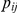 to be less than corresponding within-group link probabilities 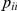 and 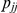. This is accomplished by adding a parameter 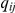 for each between-group probability, and setting 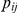 equal to 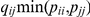.

### Markov-chain Monte Carlo Sampling

We sample from the posterior distribution of model parameters using a Markov-chain Monte Carlo technique known as Metropolis-coupled MCMC, or 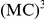 [59], which improves mixing between multiple modes of the posterior distribution, and also allows improved estimation of the marginal likelihood [60]. Software for performing MCMC sampling was implemented in Java, and is available from the corresponding author on request. A full treatment of MCMC is given in supporting text S1, section 4, including details on applying the method to the group model.

### Bayesian Model Selection

In order to select a good model variant, we employ the marginal likelihood, the probability of data given a model integrated over all model parameters (partitions and link probability parameters). This approach extends the use of Bayes' rule to model selection as well as inference of parameter values. The ratio of the marginal likelihoods for two models is often called the Bayes factor [61]–[63], and determines the posterior odds ratio of two models given equal prior odds. For details on marginal likelihood-based model selection, see text S1, section 5.

### Consensus Partitions

The output of an MCMC simulation includes a large number of network partitions representing draws from the posterior distribution. As these partitions are potentially all distinct from each other, but represent similar tendencies of species to be grouped together, it is useful to try to summarize the information contained in all the samples in a more compact form. To do this, we construct an affinity matrix with entries equal to the posterior probability that two species are grouped together. We use the affinity matrix to then form a consensus partition, using an average-linkage clustering algorithm (see supporting text S1, section 6). The affinity matrix is akin to the co-classification matrix previously used to identify uncertainty in end-points in a simulated annealing algorithm [64].

### Habitat Signature

In order to test the overall presence of habitat signature in plant groups, we assigned plants to habitat types via one of three methods based on data availability. For plants present in 133 plots sampled from around the Serengeti [65], we assigned them to the habitat type of the plot in which they were most abundant; plot habitat types were assigned via a separately compiled map of habitat boundaries [66]. Some plants were available from a study of kopje forbs [67]. Finally, some were assigned from personal knowledge of the system.

We used a randomization test to measure the overall clustering of habitat in groups across sampled partitions. The habitat signature of an individual group 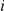 was measured as the Shannon entropy—low entropy indicates an uneven distribution—of the assignment of species to habitats, 
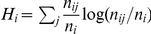
, where 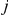 is the habitat, 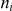 is the group size, and there are 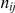 species assigned to habitat 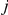 within the group. The overal clustering signature for a partition was the average of the individual group entropies, weighted by the size of the groups, 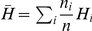, for total species count 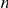. The p-value for the statistic is the probability that a partition drawn from the posterior distribution has overall clustering greater than or equal to a randomized partition with groups of identical size.

To test clustering significance of a specific habitat type in a specific grouping of species, we calculated the p-value as the probability that a randomized group of the same size would have as many or more species assigned to the chosen habitat type.

## Supporting Information

Figure S1 **Adjacency matrix ordered by groups.** Species are identically ordered top to bottom and left to right according to the consensus partition as listed in Table 2. White matrix entries indicate that the species in the column feeds on the species in the row. Columns that would indicate prey of plant groups are omitted. Note that in a modular network according to the standard definition, links would be concentrated on the diagonal of the adjacency matrix, since they occur within groups. By contrast, here links are concentrated in off-diagonal blocks.
(TIFF)

Figure S2
**Posterior distributions of link density parameters** 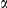 **and** 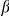 **.** Color brightness indicates posterior density, estimated using the ks multivariate kernel density estimation package for R [68]. Contours indicate cumulative density. The 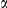 parameter is significantly lower than 1, indicating departure from a uniform distribution.(TIFF)

Figure S3
**Distribution of link probability parameters.** The prior distribution for link probability parameters, integrated over the priors for beta distribution parameters 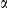 and 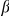, is indicated with a dotted line. The heat map shows beta distributions corresponding to the posterior distribution for 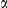 and 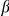, with lightness indicating the posterior density of the parameter values.(TIFF)

Table S1 **Species in the Serengeti food web.**
(CSV)

Table S2 **Feeding links in the Serengeti food web.**
(CSV)

Table S3 **Consensus partition.**
(CSV)

Table S4 **Link densities between groups in the consensus partition.**
(CSV)

Text S1 **In this supplement, we describe the mathematical details of the modeling approach, including the Bayesian formulation of the group model, the Markov-chain Monte Carlo algorithm, and marginal likelihood estimation.**
(PDF)
