# Supplementary figures and images for: Spatial Guilds in the Serengeti Food Web Revealed by a Bayesian Group Model
Source: PLoS Comput Biol. 2011 Dec 29;7(12):e1002321. doi: 10.1371/journal.pcbi.1002321 (PMC3248389; doi:10.1371/journal.pcbi.1002321)

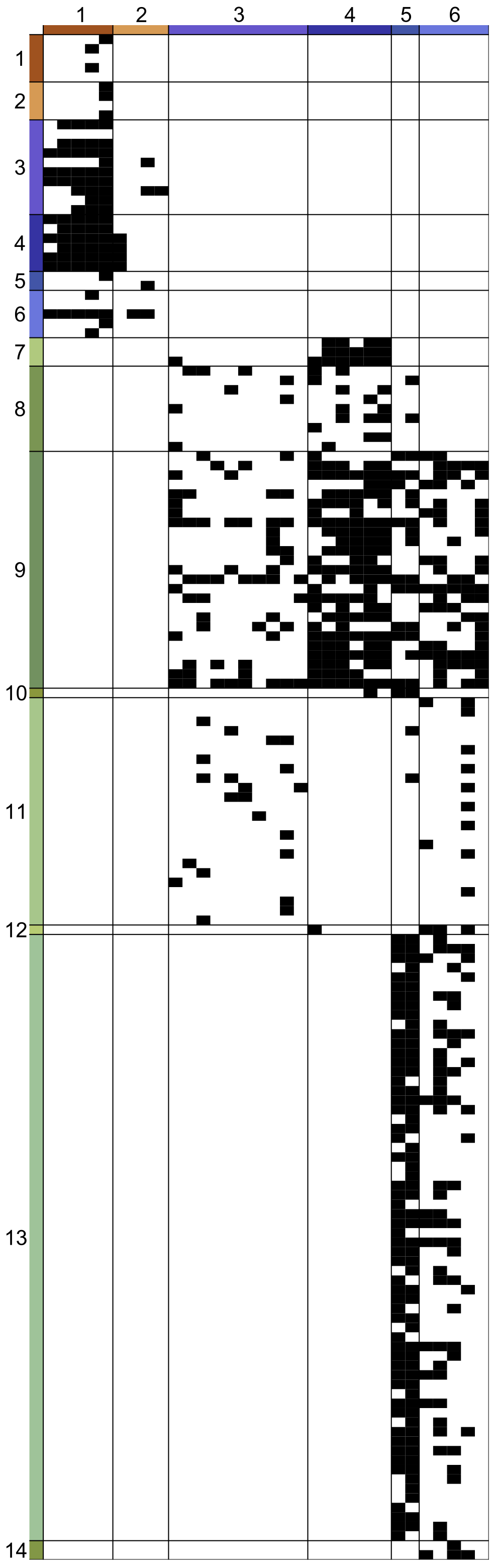

Supplement: Figure S1 — Adjacency matrix ordered by groups. Species are identically ordered top to bottom and left to right according to the consensus partition as listed in Table 2. White matrix entries indicate that the species in the column feeds on the species in the row. Columns that would indicate prey of plant groups are omitted. Note that in a modular network according to the standard definition, links would be concentrated on the diagonal of the adjacency matrix, since they occur within groups. By contrast, here links are concentrated in off-diagonal blocks. (TIFF) [file pcbi.1002321.s001.tif]

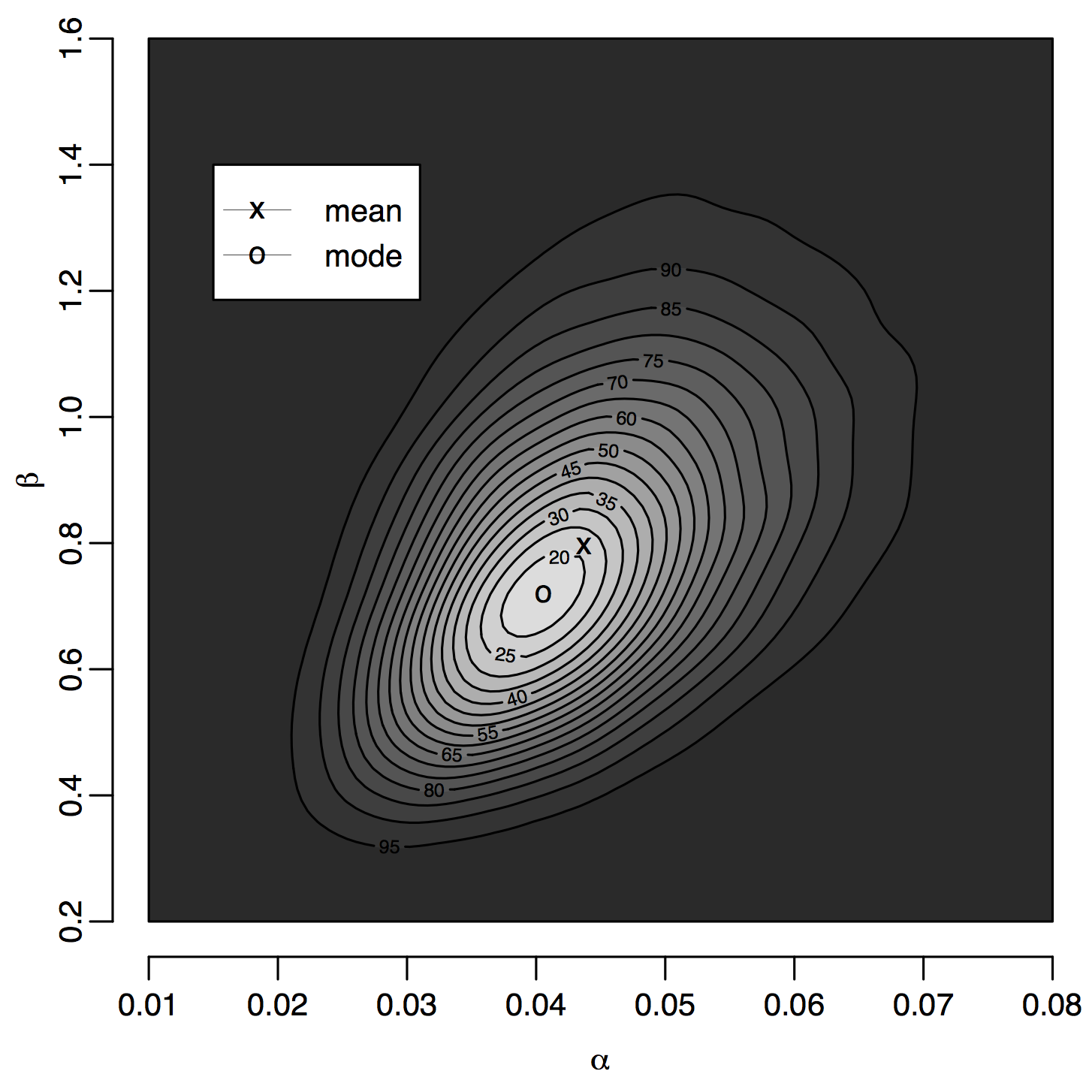

Supplement: Figure S2 — Posterior distributions of link density parameters and . Color brightness indicates posterior density, estimated using the ks multivariate kernel density estimation package for R [68]. Contours indicate cumulative density. The parameter is significantly lower than 1, indicating departure from a uniform distribution. (TIFF) [file pcbi.1002321.s002.tif]

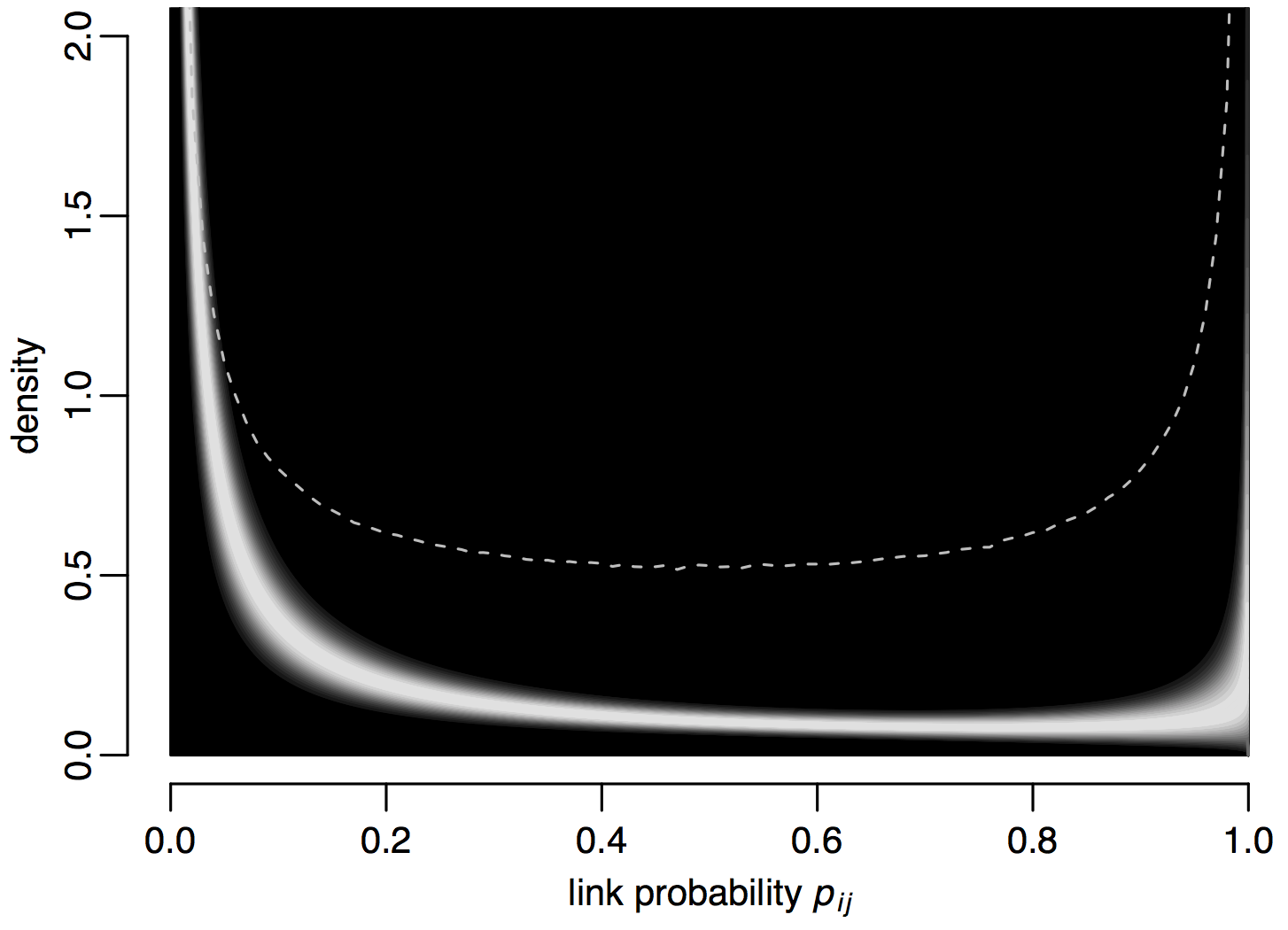

Supplement: Figure S3 — Distribution of link probability parameters. The prior distribution for link probability parameters, integrated over the priors for beta distribution parameters and , is indicated with a dotted line. The heat map shows beta distributions corresponding to the posterior distribution for and , with lightness indicating the posterior density of the parameter values. (TIFF) [file pcbi.1002321.s003.tif]
