# Supplementary material for: Spatial Guilds in the Serengeti Food Web Revealed by a Bayesian Group Model
Source: PLoS Comput Biol. 2011 Dec 29;7(12):e1002321. doi: 10.1371/journal.pcbi.1002321 (PMC3248389; doi:10.1371/journal.pcbi.1002321)
Supplement: Text S1 — In this supplement, we describe the mathematical details of the modeling approach, including the Bayesian formulation of the group model, the Markov-chain Monte Carlo algorithm, and marginal likelihood estimation. (PDF) [file pcbi.1002321.s008.pdf]

Text S1: Bayesian Inference Methods for Food-Web Models

Supplement for:
Spatial Guilds in the Serengeti Food Web Revealed by a Bayesian Group Model (Baskerville et al.)

20 November 2011

In this supplement, we describe the general Bayesian modeling approach, the mathematical details of the model, and the computational techniques used to perform inference and model selection.

1 Probabilistic Models for Food Webs

In this paper, we use probabilistic modeling as a tool for formalizing hypotheses about food web structure. We treat a food web, an observed network of who eats whom in an ecosystem, as data. We start with the basic question: assuming a probabilistic model of food web structure, what is the probability of observing this particular real-world food web? This probability is referred to as the *likelihood* of observing the data, given model parameters. In a maximum-likelihood framework, the mechanical part of the inference process is to find the set of model parameters that makes the likelihood as great as possible, with the interpretation that this represents the best point estimate of the underlying process.

We begin with the group model of Allesina and Pascual [1], which was originally treated in a maximum-likelihood framework. Conceptually, this model encodes the simple hypothesis that species can be divided into groups, and species in the same group have statistically similar behavior: they tend to consume species in certain groups and tend to be consumed by species in certain groups. Specifically, the probability that a species belonging to group i is eaten by a species belonging to group j is given by p_{ij}, and conversely, the probability of a link being absent is $(1 - p_{ij})$. If there are K groups, then a matrix $\mathbf{P}$ of K^2 link probabilities is required to completely describe the relationships among all groups. The likelihood for the whole network is the product over all pairs of species of the probability of a link being present (if present) or absent (if absent). In the statistical literature, this model structure is known as a stochastic block model [2]. The assignment of species to groups is also an unobserved parameter in this model, which adds a layer of difficulty to parameter estimation. For example, in a network of 100 species, there are approximately 5×10^{115} different ways to partition the network into groups (see Methods). That is, if you had a computer that could process 10^{80} partitions (as many partitions as there are atoms in the universe) every femtosecond (10^{-15} s), it would take 1.5×10^{13} years to process them all. (By comparison, the universe is only 1.4×10^{10} years old.)

The group model allows for a more flexible definition of groups than standard approaches to network clustering, which find groups that have large numbers of internal connections and relatively few connections between groups [3]. Because each p_{ij} parameter may take any value between 0 and 1, good model fits may result from other relationships, such as high link density between groups and low link density within groups, and may accommodate different relationships in different parts of the network. In general, the best-fitting partitions will try to maximize or minimize the number of links within specific groups and between specific pairs of groups.

2 Mathematical Formulation of the Group Model

In the group model, a network of N nodes is partitioned into K groups. The groups to which a potential prey and to which a potential predator belong completely determine the probability that a feeding relationship exists between them. The assignment of species to groups is given by the vector $\mathbf{G} = (g_1, \ldots, g_n)$, with $g_i \in \{1, \ldots, K\}$. We refer to this assignment as a set 'partition,' in keeping with standard mathematical terminology. The probability that a species assigned to group i is consumed by a species assigned to group j is equal to p_{ij}. This gives a matrix $\mathbf{P}$ of K^2 probabilities, containing the probabilities of observing directed links between members of each pair of groups, and within members of each group.

If we take $\mathbf{A}$ to be the directed adjacency matrix of a network, with entries a_{ij} equal to 1 if a link exists from node i to node j, 0 otherwise, then the probability of the network being generated by partition $\mathbf{G}$ and link probabilities $\mathbf{P}$ is given by

$$f(\mathbf{A}|\mathbf{G},\mathbf{P}) = \prod_{i=1}^{K}\prod_{j=1}^{K} p_{ij}^{Y_{ij}}(1-p_{ij})^{Z_{ij}} , \tag{1}$$

where Y_{ij} and Z_{ij} are the number of 1-entries and 0-entries in the submatrix of $\mathbf{A}$ containing entries from rows r satisfying $g_r = i$ and columns c satisfying $g_c = j$.

In the simplest case, all nodes are assigned to the same group, and the likelihood simplifies to

$$f(\mathbf{A}|p) = p^Y(1-p)^Z \tag{2}$$

where Y and Z are the total number of 1-entries and 0-entries in $\mathbf{A}$.

2.1 Compartmental modification

The group model may be restricted to compartmental partitions by requiring between-group link probability parameters to be higher than corresponding within-group parameters. A simple way to fix this requirement is by defining a new parameter q_{ij} between 0 and 1, and re-defining values of p_{ij} so that

$$p_{ij} = \begin{cases} q_{ij} & i = j \\ q_{ij}\min(q_{ii}, q_{jj}) & i \neq j \end{cases} \tag{3}$$

This way, all between-group parameters are restricted to be less than the within-group parameters for both groups.

3 Bayesian Inference and Priors for the Group Model

In a Bayesian framework, rules of probability are taken to govern both the data and model parameters. Rather than finding the set of parameter values that maximize the likelihood, the goal becomes to estimate a probability distribution over parameters based on observed data. In this way, we can directly quantify the uncertainty in our parameters in terms of probabilities. This permits questions such as: what is the probability that a parameter lies in a particular range? The name "Bayesian" comes from Bayes' rule, which tells us how to use conditional probability statements to infer a *posterior* distribution, in this case, the probability distribution over parameter values conditional on having observed the data, $\Pr(\theta|D)$. If we are dealing entirely with discrete probability distributions, Bayes' rule takes its most intuitive form:

$$\Pr(\theta|D) = \frac{\Pr(\theta)\Pr(D|\theta)}{\Pr(D)}. \tag{4}$$

The numerator of the right-hand-side is the probability of producing the data from the given parameters: the *prior* probability of those parameters, $\Pr(\theta)$, times the probability of producing the data given those parameters, $\Pr(D|\theta)$, the likelihood. The denominator is the *marginal* probability of observing the data unconditional on the particular parameter values at play, which is simply the sum of the probabilities of all the different ways of producing the data using all possible parameter values, $\Pr(D) = \sum_\theta \Pr(\theta)\Pr(D|\theta)$. In other words, in order to calculate the posterior probability of parameters θ, we add up all the different ways of producing the data weighted by their probability, and then calculate what fraction of that probability came from parameters θ. From here, we will write these quantities in more general notation, suitable for a mix of discrete and continuous probability distributions:

$$f(\theta|D) = \frac{f(\theta)f(D|\theta)}{\int_\theta f(\theta)f(D|\theta)\,d\theta} \tag{5}$$

where the integral sign represents a multiple integral over discrete and continuous parameters.

In the Bayesian framework, the model includes not only the formulation of the likelihood but also a prior distribution over parameters. With the group model, this means defining a prior distribution over both link probabilities and arrangements into groups ("partitions"). In general, priors may incorporate informed knowledge about the system, but in this case we simply use them to encode different variants of the same basic model. We use two distributions for partitions and two distributions for link probabilities, which are combined to form four different model variants.

The two alternative distributions for elements p_{ij} of the link probability matrix $\mathbf{P}$ are (1) a uniform distribution between 0 and 1, and (2) a beta distribution with shape parameters α and β, which are in turn governed by exponential distributions with mean 1. With α and β fixed at their means, alternative (2) reduces to a uniform distribution; at other values, the distribution may take a uniform, convex, concave, or skewed shape. Alternative (2) is thus structured hierarchically, with exponential *hyperpriors* for α and β governing the beta prior for elements of $\mathbf{P}$.

For partitions, we consider (1) a uniform distribution and (2) a distribution generated by the Dirichlet process, sometimes referred to as the "Chinese restaurant process" [4]. Alternative (2) is controlled by an aggregation parameter χ that is in turn drawn from an exponential distribution with mean 1. The uniform distribution assigns equal prior probability to each possible partition,

irrespective of the number of groups. Because there are far more ways to partition the network at an intermediate, but relatively high, number of groups, the uniform prior implicitly biases the model toward that number. For example, for 100 nodes, the *a priori* expectation is $K = 28$ groups. In contrast, the hierarchically structured Dirichlet process prior provides flexibility via the aggregation parameter χ. When χ is large, partitions tend to have many small groups; when χ is small, partitions tend to have fewer groups, with a skewed group size distribution.

In order to use the group model for Bayesian inference, we want to infer the posterior distribution over partitions and parameters,

$$f(\mathbf{G}, \mathbf{P}|\mathbf{A}) \propto f(\mathbf{G}, \mathbf{P}) f(\mathbf{A}|\mathbf{G}, \mathbf{P}). \tag{6}$$

This requires specifying a prior distribution over partitions $\mathbf{G}$ and link probabilities $\mathbf{P}$. We consider two priors over $\mathbf{G}$ and two priors over $\mathbf{P}$.

3.1 Priors for Partitions

The simplest prior over partitions assigns equal probability to each possible assignment of nodes into groups. For a network of N nodes, the number of possible partitions is given by the Nth Bell number,

$$\mathcal{B}(N) = \sum_{K=1}^{N} \mathcal{S}_2(N, K), \tag{7}$$

where $\mathcal{S}_2(N, K)$ is the Stirling number of the second kind, the number of ways to partition N objects into exactly K groups,

$$\mathcal{S}_2(N, K) = \frac{1}{K!} \sum_{j=0}^{K} (-1)^{K-j} \binom{K}{j} j^N. \tag{8}$$

Therefore, the prior probability of a particular partition is uniform across all possible partitions

$$f(\mathbf{G}) = \frac{1}{\mathcal{B}(N)}, \tag{9}$$

and the prior probability of having exactly K groups is

$$f(K) = \frac{\mathcal{S}_2(N, K)}{\mathcal{B}(N)}. \tag{10}$$

For partitions, the choice of a uniform prior, although simple, includes hidden assumptions. In particular, there are far more possible partitions for an intermediate number of groups than a small or large number, so the prior will implicitly bias results toward that number. For example, with 100 nodes, the distribution is peaked at $K = 28$ (Figure 1).

An alternate prior for partitioning objects into groups comes from the Dirichlet process, also known as the "Chinese restaurant process," which is becoming a standard Bayesian prior for nonparametric problems [4, 5, 6]. Consider a restaurant with an infinitely large number of infinitely large tables, all initially empty. The first patron sits alone at the first table, and subsequent patrons may either

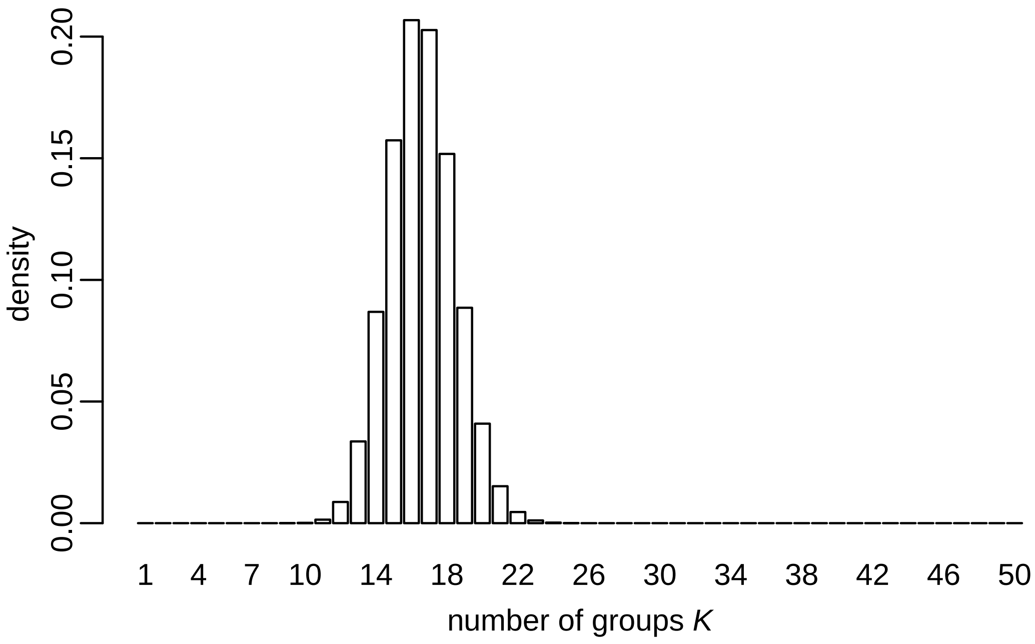

Figure 1: **Implicit prior on number of groups with uniform partition prior.** The prior distribution on number of groups K is shown for a uniform partition prior for a network with 100 nodes. The mode of the distribution is at $K = 28$.

sit at an occupied table or a new table. They choose occupied tables with weight equal to the number of current occupants, or a new table with weight equal to an aggregation parameter χ. For example, the second patron will sit at the same table as the first patron with probability $1/(1+\chi)$. In fact, because the process is *exchangeable*, the probability of any pair of patrons sitting at the table is also $1/(1+\chi)$. If χ is small, there will tend to be a small number of occupied tables and a skewed distribution of table sizes; if χ is large, there will be a larger number of tables occupied by few patrons.

Interpreting tables of patrons as groups of nodes, under the Dirichlet process the prior probability of a particular partition $\mathbf{G}$ is

$$f(\mathbf{G}|\chi) = \chi^K \frac{\prod_{j=1}^{K}(\eta_j - 1)!}{\prod_{i=1}^{N}(\chi + i - 1)}, \tag{11}$$

where N is the number of nodes in the network, K is the number of groups in the partition, and η_j is the number of nodes in group j. The prior probability of K groups is

$$f(K|\chi) = \frac{|\mathcal{S}_1(N,K)|\chi^K}{\prod_{i=1}^{N}(\chi + i - 1)}, \tag{12}$$

where $\mathcal{S}_1(N,K)$ is a Stirling number of the first kind, equal to the coefficients on x_K in the expansion $x(x-1)(x-2)\dots(x-K+1)$.

Rather than choosing a fixed value of χ for the prior, we give χ an exponential hyperprior distribution with mean 1:

$$f(\chi) = e^{-\chi} \qquad \chi \geq 0. \tag{13}$$

3.2 Priors for Link Probabilities

Similarly, the elements of link probability matrix $\mathbf{P}$ may be given a simple uniform prior over $[0, 1]$:

$$f(p_{ij}) = 1 \qquad 0 \leq p_{ij} \leq 1. \tag{14}$$

As there may be some regularity in the values of the link probabilities, we also tried a beta prior:

$$f(p_{ij}|\alpha, \beta) = \frac{1}{B(\alpha, \beta)} p_{ij}^{\alpha-1}(1 - p_{ij})^{\beta-1}, \tag{15}$$

where $B(\alpha, \beta)$ is the beta function,

$$B(\alpha, \beta) = \int_0^1 t^{\alpha-1}(1 - t)^{\beta-1}\, dt. \tag{16}$$

The parameters α and β control the shape of the distribution, which may be convex, concave, or skewed toward 0 or 1. When $\alpha = \beta = 1$, the beta prior becomes a uniform distribution.

We use α and β exponential hyperpriors with mean 1:

$$f(\alpha) = e^{-\alpha} \qquad \alpha \geq 0, \tag{17}$$

$$f(\beta) = e^{-\beta} \qquad \beta \geq 0. \tag{18}$$

4 Markov-chain Monte Carlo Sampling

For networks of any appreciable size, the number of possible partitions is far too large to enumerate, so we must use a Markov-chain Monte Carlo (MCMC) technique to sample from the posterior distribution. Here we describe the sampling procedure and the details of the Metropolis-Hastings proposal distribution used.

We employ the standard Metropolis-Hastings algorithm to sample from the posterior distribution over partitions and model hyperparameters [7, 8]. The general idea of an MCMC method is to set up a sequence of dependent samples $\theta_1, \theta_2, \ldots$ that is guaranteed to converge to a target distribution, in this case the posterior distribution of our model. Starting from the current sample, a change is proposed, drawn from a *proposal distribution* over possible changes, $q(\theta \to \theta^*)$. This sample is either rejected, in which case the current sample is repeated, or the proposed sample is accepted as the new sample. The Metropolis-Hastings acceptance probability,

$$r(\theta \to \theta^*) = \min\left\{1, \frac{f(\theta^*)}{f(\theta)} \frac{q(\theta^* \to \theta)}{q(\theta \to \theta^*)}\right\},$$

guarantees that the sequence of samples will converge to the posterior distribution, $f(\theta|D) \propto f(\theta)f(D|\theta)$, the prior times the likelihood.

For the group model, the samples θ consist of hyperparameters for the model variant—the Dirichlet process prior parameter χ and the beta prior parameters α and β—as well as the group count K and assignment vector $\mathbf{G}$. The link probabilities $\mathbf{P}$ governing links between groups are not included,

because the likelihood function would not be compatible between partitions with different values of K. One possible solution to this problem would be to include $\mathbf{P}$ in the sampling procedure, restrict K to a particular number for a particular run, and then appropriately weight runs with different values of K. Another approach is reversible-jump MCMC [9], which appropriately handles a mapping between two different parameter spaces as part of the Metropolis-Hastings proposal ratio. (We tried a reversible-jump scheme, but chains tended to get stuck at local maxima.)

Instead of trying to sample values of $\mathbf{P}$, we use the *marginal likelihood* of a partition given model hyperparameters—that is, the posterior distribution, conditional on values of α, β, and $\mathbf{G}$, integrated over all possible values of $\mathbf{P}$—directly in the Metropolis-Hastings procedure. This is possible because the marginal likelihood of a single partition can be calculated analytically.

For a beta prior over link probabilities, the likelihood of $\mathbf{G}$, α and β marginalized over all possible values of $\mathbf{P}$ is

$$f(\mathbf{A}|\mathbf{G},\alpha,\beta) = \int_{\mathbf{P}} f(\mathbf{P}|\alpha,\beta) f(\mathbf{A}|\mathbf{G},\mathbf{P})\, d\mathbf{P} \tag{19}$$

$$= \prod_{i=1}^{K}\prod_{j=1}^{K} \int_0^1 \frac{1}{B(\alpha,\beta)} p_{ij}^{\alpha-1}(1-p_{ij})^{\beta-1} p_{ij}^{Y_{ij}}(1-p_{ij})^{Z_{ij}}\, dp_{ij} \tag{20}$$

$$= \prod_{i=1}^{K}\prod_{j=1}^{K} \int_0^1 \frac{1}{B(\alpha,\beta)} p_{ij}^{Y_{ij}+\alpha-1}(1-p_{ij})^{Z_{ij}+\beta-1}\, dp_{ij} \tag{21}$$

$$= \prod_{i=1}^{K}\prod_{j=1}^{K} \frac{B(Y_{ij}+\alpha, Z_{ij}+\beta)}{B(\alpha,\beta)}. \tag{22}$$

Similarly, for a uniform prior over link probabilities, the marginal likelihood of a particular partition is simply

$$f(\mathbf{A}|\mathbf{G}) = \prod_{i=1}^{K}\prod_{j=1}^{K} B(Y_{ij}+1, Z_{ij}+1). \tag{23}$$

4.1 Proposal distribution

For the case of uniform priors on partitions and link probabilities, the proposal distribution only allows changes to the partition. With the Dirichlet process prior on partitions and the beta prior on link probabilities, hyperparameters χ, α, and β can also be changed.

The proposal distribution is described as follows:

1. Each hyperparameter h (α, β, and χ) is chosen for update with probability p_h, where p_h are tuned to improve convergence. A proposed new value h' is drawn from a uniform distribution between $\max(0, h - r_h)$ and $h + r_h$, where r_h is a proposal radius manually tuned to improve convergence. (A scale-free proposal could easily be used instead, and may require less tuning.)
2. With probability $(1 - \sum_h p_h)$, a group-change move is proposed:
   (a) A node i is chosen uniformly at random as the one to be moved.
   (b) Another node $j \neq i$ is chosen uniformly at random.

(c) If i and j are in different groups, node i is moved into the group of node j. If i and j are in the same group, node i is moved into a new group.

The proposal ratio for parameter-change proposals is 1, since a uniform proposal distribution is used, except near zero, where it becomes

$$\frac{q(h^* \to h)}{q(h \to h^*)} = \frac{h + r_h - \max(h - r_h, 0)}{h^* + r_h - \max(h^* - r_h, 0)} \,. \tag{24}$$

The proposal ratio for group-change proposals is non-uniform. If both groups are nonempty to start, N is the total number of nodes, N_i is the starting size of the group the node starts in, and N_j is the starting size of the group the node is moving to, then the probability of a forward move for any node going from group i to group j is

$$q(i \to j) = \frac{1}{N} \frac{N_j}{N-1} \,, \tag{25}$$

and the probability of the reverse move is

$$q(j \to i) = \frac{1}{N} \frac{N_i - 1}{N-1} \,, \tag{26}$$

resulting in a proposal ratio of

$$\frac{q(j \to i)}{q(i \to j)} = \frac{N_i - 1}{N_j} \,. \tag{27}$$

In the case of choosing a new group, the probability must be adjusted:

$$q(i \to j_\emptyset) = \frac{1}{N} \frac{N_i - 1}{N-1} \,. \tag{28}$$

In general, then, the proposal ratio becomes

$$\frac{q(j \to i)}{q(i \to j)} = \frac{N_r}{N_f} \tag{29}$$

where $N_f = N_j$ if the destination group j exists, $N_i - 1$ otherwise, and $N_r = N_i - 1$ if the destination group i exists (in the reverse move), N_j otherwise.

4.2 Metropolis-coupled MCMC

Although the Metropolis-Hastings algorithm is guaranteed to converge to the target distribution at some point, local maxima in the likelihood surface can cause a chain to become stuck for long periods of time. One approach to avoiding this problem, known as "Metropolis coupling," involves running multiple chains in parallel. One chain, the "cold chain," explores the target distribution, while the other chains, "hot chains," explore low-likelihood configurations more freely. Periodically, swaps are proposed between chains, allowing good configurations discovered on hot chains to propagate toward the cold chain.

Rather than exploring the target distribution $f(\theta|D) \propto f(\theta) f(D|\theta)$, heated chains explore

$$f_\tau(\theta|D) \propto f(\theta) \left[f(D|\theta)\right]^\tau \qquad \tau \in [0,1], \tag{30}$$

where τ is a heating parameter. We use linearly spaced values of τ, with the hottest chain exploring the prior ($\tau = 0$) and the coldest chain exploring the posterior ($\tau = 1$).

Swap moves are standard Metropolis-Hastings proposals, but rather than considering a change to a single chain, they consider a change to the joint distribution of two chains. The acceptance probability is thus the ratio of the joint distribution after and before the move:

$$r\left((\theta_i, \theta_j) \to (\theta_j, \theta_i)\right) = \frac{f(\theta_j)\left[f(D|\theta_j)\right]^{\tau_i} f(\theta_i)\left[f(D|\theta_i)\right]^{\tau_j}}{f(\theta_i)\left[f(D|\theta_i)\right]^{\tau_i} f(\theta_j)\left[f(D|\theta_j)\right]^{\tau_j}} \tag{31}$$

$$= \left[\frac{f(D|\theta_i)}{f(D|\theta_j)}\right]^{\tau_j - \tau_i}, \tag{32}$$

where θ_i, θ_j are the configurations that begin in chains i and j, and τ_i, τ_j are the heat parameters of the two chains.

The use of multiple heated chains has the side effect of drastically improving estimates of marginal likelihoods for model selection, as described in the next section.

5 Model Selection via Marginal Likelihood

The Bayesian framework provides a natural way to make probabilistic inferences based on a particular model. However, we also want to be able to choose between different models by quantifying their relative goodness of fit. One approach to Bayesian model selection can be framed directly in terms of Bayes' rule, mirroring the process for estimating the posterior distribution over parameters for a single model.

Consider two models, M_1 and M_2, to which we assign prior weight $\Pr(M_1)$ and $\Pr(M_2)$. After the data has been observed, we can calculate the posterior probability of the models using Bayes' rule:

$$\Pr(M_1|D) = \frac{\Pr(M_1)\Pr(D|M_1)}{\Pr(D)}, \tag{33}$$

$$\Pr(M_2|D) = \frac{\Pr(M_2)\Pr(D|M_2)}{\Pr(D)}, \tag{34}$$

where the denominator is equal to the probability of observing the data unconditional of the particular model at play, $\Pr(D) = \Pr(M_1)P(D|M_1) + \Pr(M_2)P(D|M_2)$. The probabilities $\Pr(D|M_1) = \int_{\theta_1} f(\theta_1)f(D|\theta_1)\,d\theta_1$ and $\Pr(D|M_2) = \int_{\theta_2} f(\theta_2)f(D|\theta_2)\,d\theta_2$ are the marginal likelihoods of the two models, corresponding to the denominator in Equation 5. If we give the two models equal prior weight, then the relative posterior weight of the two models is simply given by the marginal likelihoods. This reasoning extends naturally to any number of models.

The ratio of the marginal likelihoods is often called the Bayes factor [10, 11, 12], and is equal to the posterior odds ratio of the two models, assuming equal prior weight:

$$B_{12} = \frac{\Pr(D|M_1)}{\Pr(D|M_2)} \tag{35}$$

The Bayes factor provides a convenient way to compare models: if $B_{12} = 10$, then we consider support for model M_1 to be ten times stronger than model M_2. In AIC-based model selection, the Bayes factor is analogous to a ratio of Akaike weights [13].

The marginal likelihood of a model is the likelihood averaged over the prior distribution. That is, it is the likelihood one would expect by randomly sampling parameters from the prior distribution:

$$f(D|M) = \int_\theta f(\theta) f(D|\theta)\, d\theta\,. \tag{36}$$

This value serves as a useful measure of model fit because it directly incorporates the dependence of the likelihood on uncertainty in parameter values, implicitly penalizing extra degrees of freedom [14]. If an additional parameter improves the maximum likelihood but decreases the average likelihood, the model suffers from overfitting relative to the simpler model.

5.1 Marginal likelihood for the group model

The marginal likelihood for the group model involves integrating over all hyperparameters, partitions, and link probabilities. For the model with uniform distributions over partitions and link probabilities, the marginal likelihood is

$$f(\mathbf{A}|M_{u,u}) = \sum_{\mathbf{G}} f(\mathbf{G}) f(\mathbf{A}|\mathbf{G}) \tag{37}$$

$$= \sum_{\mathbf{G}} \frac{1}{\mathcal{B}(N)} \left[\prod_{i=1}^{K} \prod_{j=1}^{K} B(Y_{ij}+1, Z_{ij}+1) \right]. \tag{38}$$

With a Dirichlet process prior over partitions and a uniform distribution over link probabilities, the marginal likelihood is similarly

$$f(\mathbf{A}|M_{d,u}) = \int_0^\infty f(\chi) \sum_{\mathbf{G}} f(\mathbf{G}|\chi) f(\mathbf{A}|\mathbf{G})\, d\chi. \tag{39}$$

Using a uniform prior over partitions and a beta prior over link probabilities yields

$$f(\mathbf{A}|M_{u,b}) = \sum_{\mathbf{G}} f(\mathbf{G}) \int_0^\infty f(\alpha) \int_0^\infty f(\beta) f(\mathbf{A}|\mathbf{G},\alpha,\beta)\, d\beta\, d\alpha\,. \tag{40}$$

Combining both gives

$$f(\mathbf{A}|M_{d,b}) = \int_0^\infty f(\chi) \sum_{\mathbf{G}} f(\mathbf{G}|\chi) \int_0^\infty f(\alpha) \int_0^\infty f(\beta) f(\mathbf{A}|\mathbf{G},\alpha,\beta)\, d\beta\, d\alpha\, d\chi. \tag{41}$$

5.2 Marginal likelihood estimation

As enumeration across all possible partitions is impossible for networks of any significant size, we would like to use MCMC to estimate the marginal likelihood for the sake of comparison among different models. Marginal likelihood estimates derived from a single chain, such as the harmonic

mean estimator of Raftery [12], converge very slowly, because MCMC fails to sample sufficiently from low-likelihood areas. However, it is possible to use the information gathered about low-likelihood areas in heated chains using a technique called thermodynamic integration [15, 16], or path sampling [17].

Assuming a continuum of heated chains, the thermodynamic estimator for the log-marginal likelihood is

$$\log \hat{\mathcal{L}}(M) = \int_0^1 \frac{1}{m} \sum_{i=1}^{m} \pi(\theta_{i,\tau}) \log \mathcal{L}(\theta_{i,\tau})\, d\tau \tag{42}$$

where m is the number of samples in the MCMC output, and $\theta_{i,\tau}$ is a single sample from the output in a chain with heat parameter τ [16]. With a finite number of chains, we use the trapezoid rule to numerically integrate this integral, using uneven spacing of heats to improve the estimate [18].

6 Consensus Partitions

The output of an MCMC simulation includes a long sequence of network partitions representing draws from the posterior distribution over partitions. As these partitions are potentially all distinct from each other, but represent similar tendencies of species to be grouped together, it is useful to try to summarize the information contained in all the samples in a more compact form. One approach is to construct a pairwise "affinity matrix" for species in the food web, with entries equal to the posterior probability that two species are in the same group. A visual representation of this matrix can illuminate the group structure, and a consensus partition can then be constructed from this matrix using a simple clustering algorithm.

The full output of an MCMC chain from the group model includes an extremely large number of different partitions, and, for the sake of interpretation, it is desirable to seek a *consensus partition* that does a reasonable job of summarizing the distribution. We use a simple, computationally inexpensive method to accomplish this task: in short, clustering the nodes in the network based on a pairwise affinity matrix matrix.

The affinity matrix $\mathbf{M}$ is the posterior probability that two nodes are in the same group and 0 otherwise, that is,

$$\mathbf{M} = \sum_{\mathbf{G}} P(\mathbf{G}|\mathbf{A})\mathbf{M}_{\mathbf{G}}\,, \tag{43}$$

where an entry $\mathbf{M}_{\mathbf{G}}$ is 1 if nodes i and j are in the same group, that is,

$$\mathbf{M}_{\mathbf{G},ij} = \delta_{\mathbf{G}_i,\mathbf{G}_j}\,, \tag{44}$$

where δ is the Kronecker delta and $\mathbf{G}$ is the assignment vector for the partition. This matrix is estimated from MCMC output as the fraction of MCMC samples in which the corresponding species are in the same group:

$$\hat{\mathbf{M}} = \frac{1}{N} \sum_{i=1}^{N} \mathbf{M}_{\mathbf{G}_i}\,. \tag{45}$$

A consensus partition is formed by applying a hierarchical clustering algorithm to the affinity matrix estimate $\hat{\mathbf{M}}$, and then cutting the dendrogram at some number of groups K, forming a consensus partition with assignment vector $\mathbf{G}_K$ and affinity matrix $\mathbf{M}_K$. The goodness of fit of a consensus partition is simply measured as the correlation between $\hat{\mathbf{M}}$ and $\mathbf{M}_K$). The best consensus partition is thus identified using the value of K that gives the highest correlation.

We use the average-linkage clustering algorithm [19] as implemented by the `hclust` function in the R software package [20], treating $\mathbf{1} - \hat{\mathbf{M}}$ as distance matrix. We find that the average-linkage algorithm produces higher correlations than the other algorithms implemented as well as ideal K close to the mean K in the MCMC output. Furthermore, we find that consensus partitions produce higher correlations with the $\hat{\mathbf{M}}$ than any individual partition in the MCMC output.

References

[1] Allesina S, Pascual M (2009) Food web models: a plea for groups. Ecol Lett 12: 652–62.

[2] Wang Y, Wong G (1987) Stochastic blockmodels for directed graphs. J Am Stat Assoc 82: 8–19.

[3] Girvan M, Newman M (2002) Community structure in social and biological networks. Proc Natl Acad Sci USA 99: 7821.

[4] Ferguson T (1973) A Bayesian analysis of some nonparametric problems. Ann Stat 1: 209–230.

[5] Huelsenbeck JP, Suchard MA (2007) A nonparametric method for accommodating and testing across-site rate variation. Syst Biol 56: 975–87.

[6] Xing E, Jordan M, Sharan R (2007) Bayesian haplotype inference via the Dirichlet process. Journal of Computational Biology 14: 267–284.

[7] Metropolis N, Rosenbluth A, Rosenbluth M, Teller A, Teller E (1953) Equation of state calculations by fast computing machines. Journal of Chemical Physics 21: 1087.

[8] Hastings W (1970) Monte Carlo sampling methods using Markov chains and their applications. Biometrika 57: 97–109.

[9] Green P (1995) Reversible jump Markov chain Monte Carlo computation and Bayesian model determination. Biometrika 82: 711.

[10] Jeffreys H (1935) Some tests of significance, treated by the theory of probability. Proc Cambridge Philos Soc 31: 203–222.

[11] Jeffreys H (1961) Theory of Probability. The International Series of Monographs on Physics. Oxford: Clarendon Press, 3rd edition.

[12] Kass R, Raftery A (1995) Bayes factors. J Am Stat Assoc 90: 773–795.

[13] Burnham KP, Anderson D (2002) Model Selection and Multi-Model Inference. Springer.

[14] Bolker BM (2008) Ecological Models and Data in R. Princeton University Press.

[15] Lartillot N, Philippe H (2006) Computing Bayes factors using thermodynamic integration. Syst Biol 55: 195–207.

[16] Beerli P, Palczewski M (2010) Unified framework to evaluate panmixia and migration direction among multiple sampling locations. Genetics 185: 313–26.

[17] Gelman A, Meng X (1998) Simulating normalizing constants: from importance sampling to bridge sampling to path sampling. Statistical Science 13: 163–185.

[18] Calderhead B, Girolami M (2009) Estimating bayes factors via thermodynamic integration and population mcmc. Computational Statistics & Data Analysis 53: 4028–4045.

[19] Sokal R, Michener C (1958) A statistical method for evaluating systematic relationships. University of Kansas Science Bulletin 38: 1409–1438.

[20] R Development Core Team (2010) R: A Language and Environment for Statistical Computing. R Foundation for Statistical Computing, Vienna, Austria. URL `http://www.R-project.org`. ISBN 3-900051-07-0.
